# The regulating role of galectin-9 in immune cell populations

**DOI:** 10.3389/fphar.2024.1462061

**Published:** 2024-10-30

**Authors:** Zhanqi Cao, Ping Leng, Hanlin Xu, Xiangpeng Li

**Affiliations:** Department of Pharmacy, The Affiliated Hospital of Qingdao University, Qingdao, China

**Keywords:** galectin-9, immune, T cell, strategies, inhibitors

## Abstract

Galectin-9 (gal-9) is a protein that belongs to the galectin family. Gal-9 is expressed in cells of the innate and adaptive immune system, including lymphocytes, dendritic cells, giant salivary cells, eosinophils and T cells, etc. In different immune cells, the role of gal-9 is different. Gal-9 can induce the proliferation and activation of immune cells, and also promote the apoptosis of immune cells. This effect of gal-9 affects the occurrence and development of a variety of immune-related diseases, such as the invasion of pathogenic microorganisms, immune escape of tumor cells, and inflammatory response. Thus, understanding the biological roles of gal-9 in innate and adaptive immunity may be essential for autoimmune diseases treatment and diagnosis to improve patient quality of life. In this review, we aim to summarize current research on the regulatory roles of gal-9 in human immune system and potential inducers and inhibitors of gal-9, which may provide new strategies for immune diseases therapies.

## 1 Introduction

**FIGURE 1:**
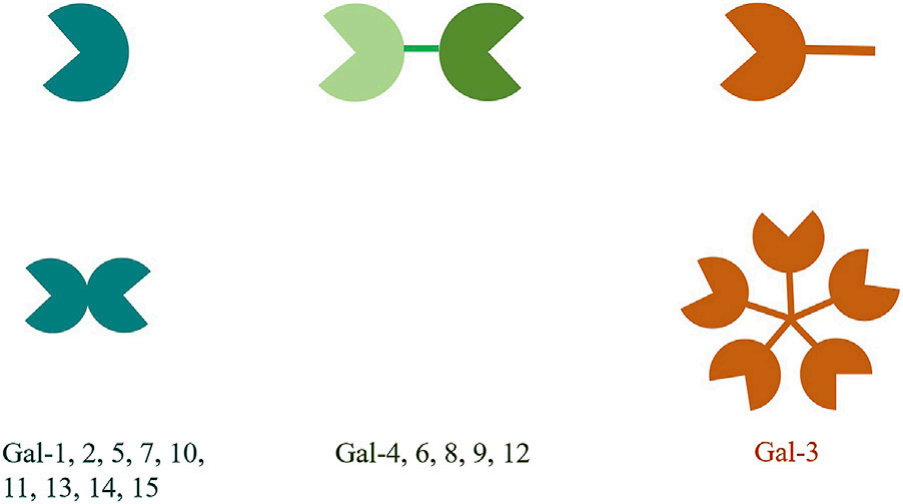
The structural classification of galectins is divided into three types. The prototype galectins possess one CRD and exist in a dimeric form. The chimeric galectin-3 has a non-CRD structural domain at the N-terminus and a CRD structural domain at the C-terminus. The non-CRD structural domain of galectin-3 participates in the assembly of pentamers. The tandem repeat galectins have two CRDs that are connected by a short linker peptide.

The galectin (gal) family is a family of endogenous lectins with high affinity to polyglycans containing galactose residues, belonging to the animal lectins in the lectin family. To date, 15 members have been discovered and named, all of which have one or two carbohydrate recognition domains (CRDs) and have a special affinity for β-galactoside (Leffler et al., 2002). According to the structure and the number of the CRDs, the gals can be divided into 3 types. The first type is called prototype gal, which only has a CRD and can form a non-covalent homodimer, including gal-1, -2, -5, -7, -10, -11, -13 -14 and -15. The second type is tandem repeat gal, which has two similar CRDs, including gal-4, -6, -8, -9 and -12. The third type is gal-3, which is a chimeric gal that contains a CRD and an elongated N-terminal region domain (NRD) (Figure 1). Gals can be expressed in cytoplasm, nucleus, cell membrane or extracellular matrix, and can participate in cell growth and differentiation, cell signal transduction, cell apoptosis, secretion of inflammatory factors, immunomodulation and tumor progression, etc., (Arthur et al., 2015).

Gal-9 was first isolated from mouse fetal kidney tissue in 1997 (Wada and Kanwar, 1997). Later, Gal-9 was gradually found to be widely distributed in liver, pancreas, spleen, heart, small intestine, lung and other tissues and organs, and has a wide range of biological activities, such as participating in cell differentiation and maturation, regulating cell chemotaxis, aggregation and adhesion, and inducing cell apoptosis (Rabinovich and Toscano, 2009; Schnaar, 2016; Golden-Mason and Rosen, 2017). Gal-9 can be found in the cytoplasm, cell surface and circulation. But the source of gal-9 in the serum has not been fully identified. A small amount of literature suggests that gal-9 in serum may be released by immune cells and endothelial cells, while tumor cells lack the ability to release gal-9 (Hirashima et al., 2002; Iqbal et al., 2022; Reyes-Vallejo et al., 2022). A growing body of research describes gal-9 as an independent biomarker for multiple autoimmune diseases, such assystemic lupus erythematosus (SLE) (Matsuoka et al., 2020), autoimmune hepatitis (AIH) (Matsuoka et al., 2019), systemic sclerosis (SSc) (Chihara et al., 2018), multiple sclerosis (MS) (Burman and Svenningsson, 2016), primary Sjogren’s syndrome (pSS) (van den Hoogen et al., 2020), and juvenile dermatomyositis (JDM) (Bellutti Enders et al., 2014; Wienke et al., 2019). In acute viral hepatitis, viral myocarditis and other diseases, gal-9 can reduce acute inflammatory response by promoting the proliferation of regulatory T cells and alernatively activated Th2 cells (Lv et al., 2011; Lv et al., 2012). In addition, gal-9 can also influence the prognosis of many cancers by modulating immune homeostasis (Lv et al., 2022). These data indicate that the expression of gal-9 can influence various disease through regulating immune system, which suggest that gal-9 may be a biomarker in immune disease. The current review will summarize the regulatory roles of gal-9 in regulating the function of various immune cells in immune system, which is essential for immune diseases therapies. In order to obtain more studies on gal-9 regulation of immune cell function, we conducted literature searches using PubMed, Embase and Web of Science databases. We first conducted a literature search using the subject term “galectin-9” combined with free words (various immune cells), and then selected included studies by reading literature abstracts in order to describe in more detail the regulatory role of galectin-9 in immune cells.

## 2 Gal-9 and innate immune cells

### 2.1 Gal-9 and NK cells

**FIGURE 2:**
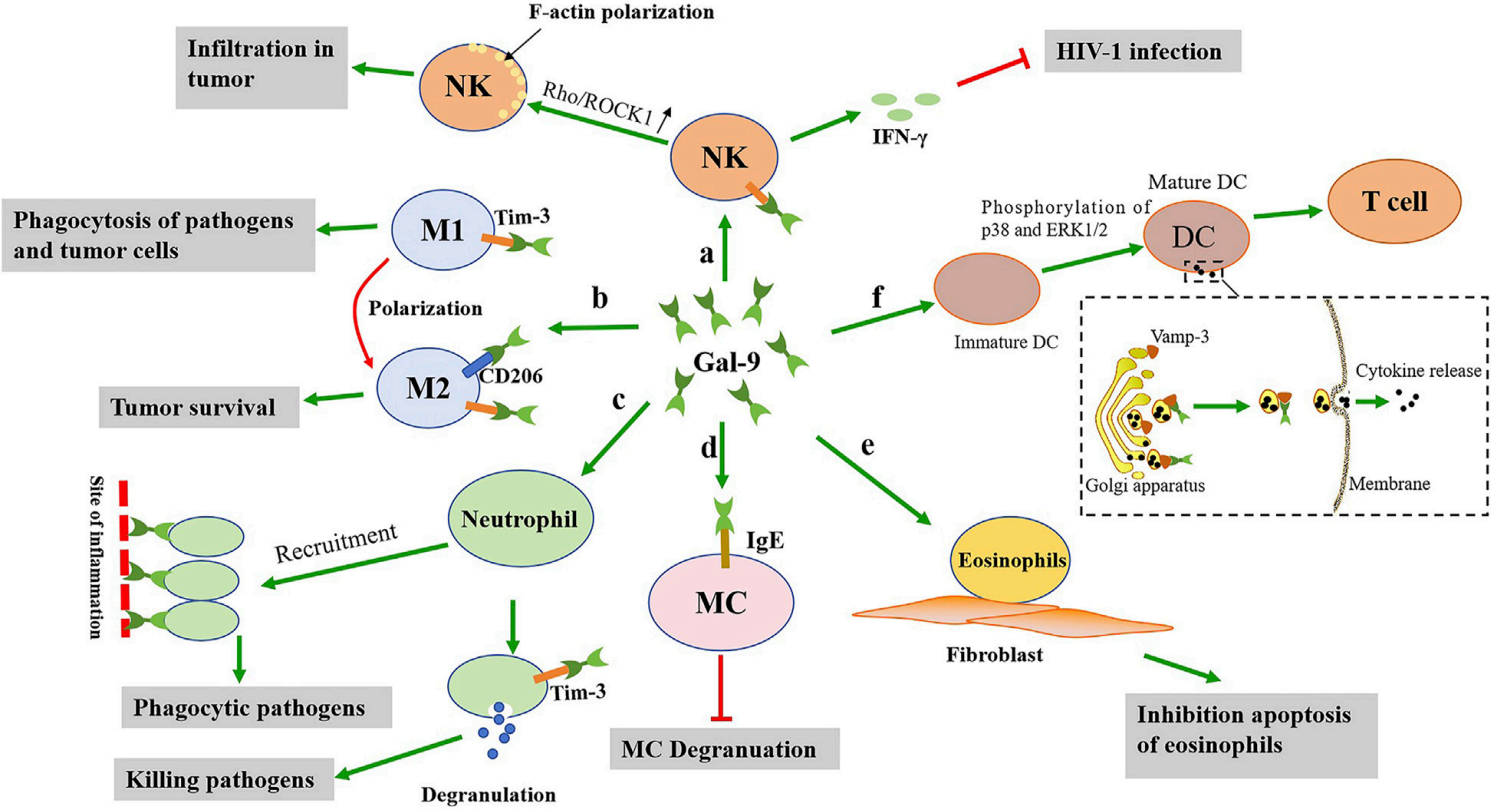
The possible effects of gal-9 on the activation of the innate immune cells. a: Gal-9 not only promotes the activation of NK cells to release IFN-γ, but also promotes the polarization of F-actin of NK cells. b: Gal-9 promotes the polarization of M1 macrophages towards M2 macrophages, which is detrimental to clear pathogens. c: Gal-9 promotes neutrophils recruitment to the site of inflammation and neutrophils degranulation, thereby clearing pathogens. d: Gal-9 binds to IgE on mast cells, and this interaction prevents the formation of IgE antigen complexes and reduces allergic reactions. e: Gal-9 can promote the adhesion of eosinophils to fibroblasts and prolong their function. f: Gal-9 promotes DCs maturation, thereby activating T cells. In addition, gal-9 promotes cytokine release by interacting with Vamp-3.

Natural killer (NK) cells are important immune cells of the innate immune system in the body, which are not only related to anti-tumor, antiviral infection and immune regulation, but also participate in the occurrence of hypersensitivity and autoimmune diseases in some cases. More and more researchers have confirmed that gal-9 plays an important role in NK cells activation and release of interferon (IFN). A previous study found that when NK92 cell lines transfected with Tim-3 were co-cultured with soluble gal-9 or Raji cell lines transfected with gal-9, a large amount of IFN-γ was produced (Gleason et al., 2012). Tim-3^+^ NK cell lines stimulated by low dose of interleukin-12 (IL-12) and IL-18 can also cause significant increase in IFN-γ secretion after interaction with soluble gal-9 or Raji cells transferred to gal-9, but the secretion of IFN-γ is significantly reduced after the addition of Tim-3 blocking antibodies (Gleason et al., 2012). This data shows that gal-9 can activate NK cells through binding with Tim-3 (Figure 2). Jost et al. (2013) found that high expression levels of gal-9 during early HIV-1 infection can lead to enhanced NK cell activity, which may improve early control of HIV-1. However, persistent gal-9 production might impair Tim-3 activity and lead to NK cell dysfunction in chronic HIV-1 infection. In another study, gal-9 was significantly upregulated on NK cells in HIV-infected groups compared to healthy controls, associated with impaired expression of cytotoxic effecting molecules granzyme B, perforin, and granin, and conversely, significantly increased expression of IFN-γ in NK cells of HIV-1-infected individuals (Motamedi et al., 2019). Carreca et al. (2022) found that IFNα-activated NK cells secrete large amounts of gal-9 and IFN-γ, which in turn inhibit HCV binding to host cells and downstream infection. Additionally, extracellular gal-9 might reduce the infectivity of hepatitis C virus by binding to the viral surface envelope protein E2. This binding may not only prevent reinfection of target cells, but may also inhibit HCV’s connection to receptors on the surface of NK cells thus interfering with the virus’s damage to effector cells (Carreca et al., 2022). However, another research showed that the expression of gal-9 could downregulate multiple immune-activating genes in NKs independent of Tim-3, which impaired the killing of lymphokine activation, and reduced the proportion of IL-12/IL-15-stimulated NK cells that produce IFN-γ (Golden-Mason et al., 2013). Moreover, in the case of short-term mouse cytomegalovirus (CMV) infection, gal-9 gene knockout caused the accumulation of eventually differentiated NK cells in the mouse liver, while the liver NK cells spontaneously produced more IFN-γ (Golden-Mason et al., 2013).

In addition, gal-9 has been found to play a positive role in the anti-tumor process by promoting the activation of NK cells (Figure 2). Multiple models and experiments have shown that NK cells can distinguish between abnormal and healthy cells, resulting in more specific anti-tumor cytotoxicity and can reduce off-target complications (Vivier et al., 2012; Guillerey et al., 2016). However, it was been reported that high infiltration or activity of NK cells in tumor is associated with better clinical outcomes in patients, depending on the cytotoxic effect of NK cells on tumor cells (Coca et al., 1997; Takeuchi et al., 2001; Li et al., 2021). In tumor-bearing mice, gal-9 increased the number of NK cells in peritoneal exudation, suggesting that gal-9 may be involved in tumor progression by regulating NK cells (Nobumoto et al., 2009). In addition, reduced expression of gal-9 in colon tumor tissues is associated with poor prognosis in these patients (Wang et al., 2016). Gal-9 could enhance F-actin polarization by activating the Rho/ROCK1 pathway of NK cells, and then promote the chemotactic movement of NKs (Wang et al., 2016). However, there is another idea that the Tim-3/gal-9 pathway mediated immune checkpoint mechanism might inhibit NK cell function in gastrointestinal stromal tumors (GIST) tissues, and this inhibition might be achieved by inhibiting or activating a complex mosaic of receptors, possibly depending on signal strength (Komita et al., 2015). Thus, Tim-3/gal-9 interaction seems to be complicated.

### 2.2 Gal-9 and macrophages

Macrophages are derived from circulating monocytes and are widely distributed in tissues and organs throughout the body, which can phagocytic and kill intracellular parasites, bacteria, tumor cells, as well as their own aging and abnormal cells. Both human and mouse macrophage subpopulations expressed high levels of gal-9, and the expression was significantly increased after proinflammatory stimulation (Krautter et al., 2020). The increased expression will then affect the immune function of the macrophages. For example, gal-9 could upregulate FcγRIIb and downregulate FcγRIII expression in macrophages and thus suppressed pro-inflammatory cytokine production and complement component C5a generation resulting in the suppression of immune complexes-mediated inflammation (Arikawa et al., 2009). Yu et al. (2018) found that *Salmonella Typhimurium* infection enhanced the expression of Tim-3 on CD4+ T cells and gal-9 on F4/80+CD11c+ macrophages in intestinal lamina propria, respectively. Through the interaction of gal-9 and Tim-3, these macrophages and CD4^+^ T cells trigger the activation of inflammasome and promote the production of IL-1β, thereby enhancing the antibacterial function of macrophages. Gal-9 expression was increased during the differentiation of monocytes into macrophages (Harwood et al., 2016). The expression of gal-9 can be involved in regulating the polarization of classical activated (M1) and alternatively activated (M2) macrophages, thereby affecting infection-related complications and their severity (Zhang et al., 2019). In the inflammatory state, macrophages are activated and differentiate into M1 and M2 macrophages. During the development of inflammation, the two macrophage phenotypes are in a state of homeostasis and are influenced by different cytokines and signaling pathways. M1 macrophages promote inflammatory responses and clear pathogens, while M2 macrophages have anti-inflammatory effects, limiting excessive inflammatory responses and promoting tissue repair (Yunna et al., 2020). The exogenous gal-9 protein could alleviate LPS-induced preeclampsia damage in rats, which may be related to macrophage metastasis to M2 subtype (Li et al., 2019).

Moreover, gal-9 also plays an important role in tumors via affecting polarization of macrophages (Figure 2). Li C. et al. (2023) knocked down the expression of gal-9 in glioblastoma cells by using T7 peptide-modified exosomes loaded with gal-9 siRNA (T7-exO/siGalectin-9), thereby activating the TLR7-IRF5 pathway. This pathway polarized macrophages to M1 phenotype, thereby enhancing macrophage phagocytosis of GBM cells. In addition, monoclonal antibody (mAb) (clonal P4D2) binding to CRD at the C-terminal of gal-9 demonstrated a unique activation property leading to malignant mesothelioma (MM) cell apoptosis, and tumors from P4D2-treated mice showed reduced tumor-associated M2 macrophage infiltration (Bertino et al., 2019). As is known, the tumor-associated macrophages (TAM) are dominated by M2-like phenotypes and expressed high levels of Tim-3 (Wang et al., 2017; Zhao et al., 2020). Therefore, the combination of gal-9 and Tim-3 may downregulate the polarization of mononuclear/macrophage cells, affect the inflammatory immune response, and downregulate the secretion of cytoplasmin and chemotactic cytokine (Zhang et al., 2019; Li et al., 2019). In PTEN-deficient GBM cells, gal-9 expression correlates with enhanced AKT activity, which inhibits IRF1 degradation by phosphorylating GSK3β into the inactivated form, thereby increasing gal-9 transcription (Ni et al., 2022). The gal-9 and Tim-3 interaction could then increase the vascular endothelial growth factor A (VEGFA) secretion by tumor-associated macrophages, thus enhancing angiogenesis (Ni et al., 2022). In addition to Tim-3, gal-9 could also bind to other partners on the membrane of the M2 macrophages. Gal-9 was found to bind to CD206 on M2 macrophages, altering the phenotype and promoting tumor survival. CD206 is a marker of M2 macrophages and plays an important role in wound healing and promoting tumor progression in tissue injury or chronic infection (Ezekowitz and Gordon, 1984; Shen et al., 2016). Moreover, another study showed that dectin-1 on macrophages in pancreatic ductal adenocarcinoma (PDA) could bind to gal-9, but inhibition of this interaction produces a strong antitumor response (Daley et al., 2017). When dectin-1 signaling was removed, the latter PDA mouse model also showed reduced tumor invasion of CD206+ macrophages. Therefore, both dectin-1 and CD206 may be involved in the regulation of macrophage function by gal-9. However, there is a different view that in tumor-bearing mice, gal-9 could induce the accumulation of unique macrophages expressing a plasmacytoid DC (pDC) -like phenotype, and then enhance the antitumor activity of NK cells (Nobumoto et al., 2009). This suggests that macrophages regulated by gal-9 have different roles in the tumor immune environment.

### 2.3 Gal-9 and neutrophil

Neutrophils are a kind of white blood cells with strong chemotaxis and phagocytosis function, accounting for 50%–70% of the total white blood cells, and the proportion of neutrophils increases significantly when inflammation occurs in the body. Its phagocytic objects are mainly bacteria, but it will also engulf other pathogens. Gal-9 acts as an adhesion molecule that captures and immobilizes neutrophils under physiological level flow and promotes recruitment of neutrophils to the site of inflammation (Iqbal et al., 2022). Under tumor necrosis factor-alpha (TNF-α) and IFN-γ stimulation, endothelial cells express and release large amounts of gal-9 into the extracellular environment (Iqbal et al., 2022; Imaizumi et al., 2002). Subsequently, gal-9 may enhance the adhesion of neutrophils to the endothelium by interacting with β2 integrin and CD44, thereby inhibiting the crawling of neutrophils on intercellular adhesion molecule-1 (ICAM-1) (Iqbal et al., 2022). By interacting with TIM-3, gal-9 induces neutrophil degranulation, promoting the killing of gram-negative bacteria (Vega-Carrascal et al., 2014). In addition, gal-9 can effectively activate neutrophil-mediated cancer cell phagocytosis, thereby eliminating epithelial cancer cells. This prophagocytosis is due to gal-9-mediated neutrophil activation, upregulation of adhesion markers, and mobilization of gelatin enzymes, secretions, and specific particles (Ustyanovska Avtenyuk et al., 2021). However, the regulatory effect of gal-9 on neutrophils is not very thorough and further studies are needed.

### 2.4 Gal-9 and dendritic cells

Dendritic cells (DCs) are the most functional professional antigen-presenting cells in the body, which can efficiently take up, process and present antigens. Immature DC has strong migration ability, and mature DC can effectively activate initial T cells, and is in the center of initiating, regulating and maintaining immune response. Gal-9 plays an important role in the maturation, stability and antigen presentation function of DCs. An early research found that gal-9 could induce monocyte derived DC maturation by promoting phosphorylation of p38 and ERK1/2 (Yamauchi et al., 2005). Since gal-9 mutants with β-galactosid-binding activity can also play this role, these effects of gal-9 on immature DCs are not primarily dependent on its lectin properties (Yamauchi et al., 2005). Moreover, gal-9 could also induce aldehyde dehydrogenase (ALDH) activity in DC by promoting the activation of p38 and PI3K signals, thereby affecting the innate immune response (de Kivit et al., 2017). A recent study has shown that gal-9 promotes the transport and secretion of intracellular cytokines such as TNF-α, IL-6, IL-10, and IL-12 through functional interactions with the vesicle-associated membrane protein (Vamp-3) (Santalla Méndez et al., 2023). Among these factors, IL-10 is a key cytokine in generating and maintaining immune tolerance (Raeiszadeh Jahromi et al., 2014), and the presence of gal-9 in conjunction with the inhibition of NF-κB is conducive to the expression of IL-10 in tolerant DC (Li L. et al., 2023). IL-10-producing DCs have the ability to regulate their immune system and can induce type 1 regulatory T cells (Tr1 cells) (Rachid and Umetsu, 2012). Intracellular vesicle transport is essential for cell homeostasis by influencing cytokine secretion and immune response initiation of DCs. Gal-9 deficient DCs accumulate cytokine-containing vesicles in the Golgi complex and eventually undergo lysosomal degradation (Santalla Méndez et al., 2023). The intracellular gal-9 controlled the aggregation of cortical actin by interacting with C-type lectin phagocytosis receptors and regulating Rac1 activity, thereby maintaining the integrity of DCs plasma membrane and pathogen uptake function (Querol Cano et al., 2019). Gal-9 could increase the number of mature DCs *in vitro* and *in vivo* (Nagahara et al., 2008), and enhance infiltration in colorectal cancer (CRC) (Wang et al., 2022). Tim-3(+) DC of tumor-bearing mice treated with gal-9 increased the number of IFN-γ-producing CD8(+) T cells, thereby enhancing the anti-tumor immunity mediated by CD8(+) T cells (Nagahara et al., 2008). These data suggests that both intracellular and extracellular gal-9 can regulate the immune function of DCs.

### 2.5 Gal-9 and mast cells

Mast cells (MCs) originate from bone marrow pluripotent hematopoietic stem cells and are distributed in connective tissue or mucosal tissue. They are effector cells of IgE-mediated rapid-onset allergy and key cells in various types of inflammatory processes. Experimental studies in mice have found that treatment with gal-9 before antigen stimulation reduces allergic airway inflammation induced by ovalbumin and mite allergens and passive skin allergic reactions after antigen stimulation (Katoh et al., 2007; Niki et al., 2009). This is because gal-9 strongly and specifically binds IgE (a highly glycosylated immunoglobulin), and this interaction prevents the formation of IgE antigen complexes and thus exerts anti-degranulation effects (Niki et al., 2009). Moreover, the presence of gal-9 could remove IgE even when IgE has been bound to mast cells and inhibits antigen-induced mast cell degranulation (Mizuno et al., 2020). The oral administration of algin increased the expression of gal-9 in colonic epithelial cells and inhibited hypersensitivity symptoms induced by mast cell activation in ovalbumin (OVA)-induced allergic response model mice (Mizuno et al., 2020). However, treatment with gal-9 after antigen attack may exacerbate inflammation in advanced stages of allergic disease by enhancing the production of cytokines and chemokines in mast cells (Kojima et al., 2014). Gal-9 may play a dual role in the function of the mast cells. Therefore, gal-9 may be a potential therapeutic target for immediate allergic reactions caused by MC degranulation (Figure 2).

### 2.6 Gal-9 and eosinophils

Eosinophils are differentiated white blood cells derived from hematopoietic stem cells, which have a role in killing bacteria and parasites, and are also extremely important cells in the process of immune response and allergic reaction. Eosinophils can release the contents of the particles, causing tissue damage and promoting the progression of inflammation. Gal-9 is considered to be an eosinophilic chemotactic agent (Matsumoto et al., 1998; Hirashima, 2000). The N-terminal and C-terminal CRD of gal-9 showed comparable eosinophilic chemotactic activity (ECA), which was much lower than that of full-length gal-9 (Hirashima, 2000). Gal-9 may induce dimerization/polymerization of cell surface glycoproteins/receptors by binding to eosinophilic surface glycoproteins, thus initiating intracellular signaling pathways associated with ECA (Sato et al., 2002). Gal-9 can promote the adhesion of eosinophils to fibroblasts, facilitate the stay of eosinophils in tissues, and prolong the effect of its function (Asakura et al., 2002). In addition, studies have shown that gal-9 not only has eosinophilic chemotactic activity, but also has several unique effects on eosinophils, such as eosinophile activation, superoxide production, and prevention of eosinophile apoptosis (Matsumoto et al., 2002). It was found that gal-9 inhibited eosinophilic apoptosis in patients with hypereosinophilic disease, while promoting eosinophilic apoptosis in healthy volunteers (Saita et al., 2002). Conversely, gal-9 accelerated Fas induced apoptosis of both types of eosinophils (Saita et al., 2002). These data suggest that gal-9 may have a heterogeneous effect on eosinophils (Figure 2).

## 3 Gal-9 and adaptive immune cells

### 3.1 Gal-9 and B cells

B cells are pluripotent stem cells derived from bone marrow. Stimulated by antigens, B cells will proliferate and differentiate into a large number of plasma cells, which can synthesize and secrete antibodies and circulate in the blood to play the function of humoral immunity. Gal-9 is considered to be an suppressor of B cell proliferation and activation (Smith et al., 2021; Ungerer et al., 2014). Gal-9 was shown to directly regulate BCR and different TLRs by binding to IgM-BCR and CD5 on the surface of B-1a cells, as well as TLR4 and the regulatory molecule CD180, and inhibit signal transduction through altering their nanoscale co-distribution (Smith et al., 2021). In the absence of gal-9, the activation of B-1a cells is enhanced. However, this enhanced activation of B-1a cells is harmful because it can promote the transfer of autoantigens to secondary lymphatic organs and exacerbate autoimmunity, thereby exacerbating the autoimmune response (Smith et al., 2021). After Epstein-Barr virus (EBV) infection of primary B cells, the expression of gal-9 continues to increase from the early stage of infection to the mature lymphoblastoid cell line (LCL) stage (Xu et al., 2023). Upregulation of gal-9 expression promoted the formation of EBV-positive B-cell lymphoma (BCL), which was associated with inhibition of STING signaling. Moreover, gal-9 level was positively correlated with disease stage and EBV nuclear antigen 1 (EBNA1) expression in BCL patients (Xu et al., 2023).

### 3.2 Gal-9 and T cells

T cells are derived from bone marrow hematopoietic stem cells in the embryonic period and grow and mature in the thymus. When external pathogens invade the body, T cells play a role in the process of fighting pathogens. T cells can be divided into helper T cells (Th), cytotoxic T lymphocyte (CTL) and regulatory T (Treg) cells according to their function, which play different roles in the adaptive immune system. Gal-9 has an inhibitory effect on the function and proliferation of T cells. By binding to Tim-3, gal-9 can interact to inhibit Th1 production by inhibiting the expression of IFN-c and IL-2 (Kuchroo et al., 2006). Activation of the gal-9/Tim-3 signaling pathway can regulate the dynamic balance of immune cells and their inflammatory response, inhibit CD4^+^ and CD8^+^ T cells, and in the process generate inhibitory signals and induce apoptosis of Th1 cells (Wang et al., 2008). Currently, gal-9 may regulates the inflammatory response of Th1 cells in two ways. One of the regulation modes is the activation of caspase-1 by gal-9, leading to inflammatory apoptosis of caspase-1 (Kashio et al., 2003). Another way is that gal-9/Tim-3 regulates T cell death by inhibiting CD11b^+^Ly-6G^+^ produce (Dardalhon et al., 2010).

**FIGURE 3:**
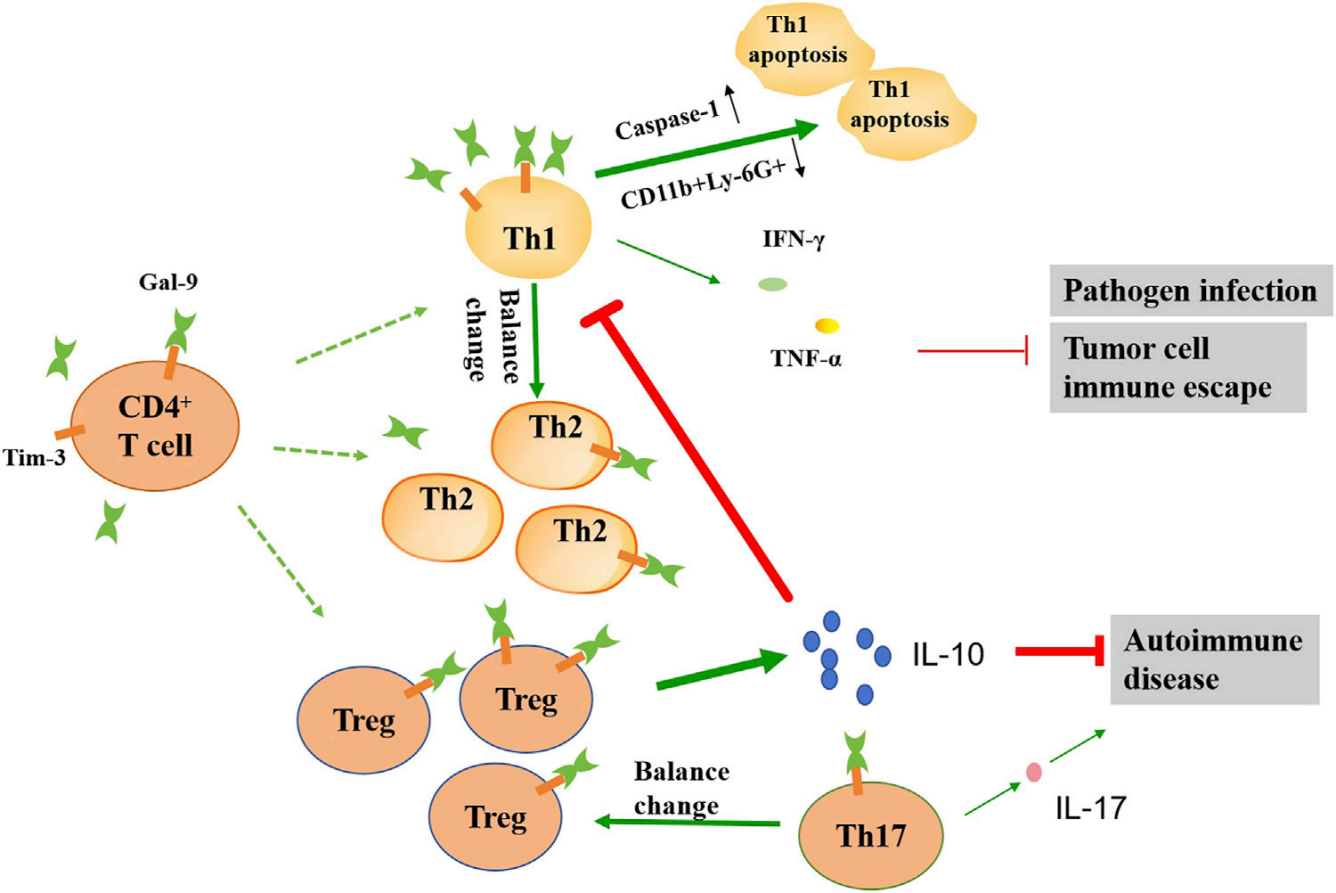
Schematic diagram illustrating the effects of gal-9 on the function of the CD4^+^ T cell subsets. In the presence of gal-9, the balance of the number of Th1, Th2, Th17 and Treg cells changes. Gal-9 can promote apoptosis of Th1 cells by up-regulating caspase-1 and down-regulating CD11b^+^Ly-6G^+^. The reduction of Th1 cells leads to reduced IFN-γ and TNF-α release, which in turn exacerbates pathogen infection and tumor progression. Gal-9 promotes the release of large amounts of IL-10 by Treg cells, which can further inhibit Th1 activation, as well as alleviate autoimmune diseases. Gal-9 can also reduce the IL-17 release by inhibiting the activation of Th17 cells, which alleviates autoimmune disease. The thickness of the line represents the strength of the promoting or inhibiting effect.

In addition, gal-9 plays a favorable role in some T-cell-related immune diseases. For example, gal-9 inhibits the development of a mouse collagen-induced arthritis (CIA) model by inducing Treg cell production while inhibiting Th17 cell production, suggesting that gal-9 improves autoimmune arthritis by regulating Treg/Th17 differentiation and Th1/Th2 imbalance (Seki et al., 2008). The balance of these types of T cells affects the development of autoimmune diseases. The cytokines secreted by Th17 cells have pro-inflammatory effects, and the cytokines secreted by Treg cells have anti-inflammatory effects. The increase of Th17 cells and the decrease of Treg cells can lead to the destruction of articular cartilage and bone, leading to rheumatoid arthritis (RA) (Lee, 2018). In a recent study, knockdown of gal-9 inhibited TNF-α activation via affecting PI3K/AKT/mTOR pathway, thereby alleviating RA (Jia et al., 2024). In experimental antiglomerular basement membrane glomerulonephritis (anti-GBM GN), Tim-3 was observed to be upregulated in the kidneys and lymph nodes, and blocking Tim-3 enhanced T-cell-mediated immunity and aggravated nephritis (Schroll et al., 2010). In addition, treatment of gal-9 reduced urinary protein excretion and crescent cell formation in rat nephrotoxic serum nephritis. These benefits were associated with apoptosis of activated CD8^+^ T cells in the spleen, but not CD4+ T cells (Tsuchiyama et al., 2000). Treatment of gal-9 reduced the proportion of CD4^+^Tim-3^+^T cells in the spleen and kidneys, and transferred Th1 to Th2, thereby reducing the immune response in anti-GBM GN (Zhang et al., 2014). A recent study shows that exogenous gal-9 can reverse Treg and effector T cell imbalance by inhibiting the PI3K/AKT signaling pathway, thereby alleviating acute graft-versus-host diseasea (GVHD) (Pang et al., 2024). Therefore, gal-9 may be a potential immunotherapeutic target for the treatment of T-cell-associated immune diseases (Figure 3).

Gal-9 can aggravate pathogen infection and tumor progression through its immunosuppressive effect. It was found that blocking the gal-9/Tim-3 signaling pathway can significantly reduce the suppressor effect on Th1 cells and increase the levels of inflammatory factors such as IFN-γ, TNF-α, IL-2 and IL-22, thereby enhancing the body’s resistance to tuberculosis (Kang et al., 2020). Blocking gal-9/Tim-3 can increase the number of effector and memory CD8^+^ T cells, thereby more effectively controlling virus infection (Sehrawat et al., 2010). In hepatitis B virus (HBV)-associated hepatocellular carcinoma (HCC), Tim-3+ CD4+ T cells express aging markers and their proliferative capacity and effector function are decreased compared with Tim-3^−^ T cells (Li et al., 2012). Tim-3/gal-9 signaling pathway mediates functional inhibition and senescence of T cells in HBV-associated HCC patients and predicts poor prognosis. In the chronic lymphocytic leukemia (CLL) tumor microenvironment, gal-9 binds to Tim-3 on the surface of Th1 and Treg cells, leading to Th1 cell depletion. At the same time, it causes excessive proliferation and activation of Treg cells, and secretes a large amount of IL-10, inhibits the ability of Th1 cells to secrete IFN-γ and TNF-α, and finally promotes the progression of CLL (Pang et al., 2021). In acute lymphoblastic leukemia (ALL), gal-9 induced apoptosis in ALL cells in a concentration-dependent manner through mechanisms associated with Bax/Bcl-2 expression and caspase-3 activation (Zargar Balajam et al., 2021). Gal-9 can induce CD8+ T cells depletion by binding with Tim-3, and promote the immune escape of diffuse large B-cell lymphoma (DLBCL) (Zhu et al., 2024). These data indicate that the gal-9 expression is related to tumor progression.

## 4 Targeted therapy for gal-9

As the expression of gal-9 can affect the progression of many autoimmune diseases by regulating the immune system. Therefore, targeted therapy against gal-9 may be a new strategy for the treatment of immune diseases. To date, many researchers have made efforts to develop ideal drugs to change the expression of gal-9 or inhibit the gal-9/Tim-3 signaling pathway, thereby improving immune diseases. The effects of several inducers or inhibitors of gal-9 on immune diseases are shown in Table 1.

**TABLE 1:** Several inducers or inhibitors of gal-9 for immune diseases.

Name	Regulatory mechanism	Disease model	Outcomes	Ref
Fucoidan	Increase gal-9 expression	Allergy model in mice	Inhibition of the decrease in rectal temperature induced by mast cell activation	Mizuno et al. (2020)
GF/Bb	Increase gal-9 expression	Allergy model in mice	Reduction of mast cell degranulation	de Kivit et al. (2012)
IFN-α	Increase gal-9 expression	Hepatoma cell line infected with the HCV JFH1 genome	IFNα-NKs secrete high levels of gal-9 neutralizing virus	Carreca et al. (2022)
Recombinant gal-9	Supplement of gal-9	Arthritis model in mice	Regulating FcγR expression on macrophages to inhibit IC-induced inflammation	Arikawa et al. (2009)
T7-Exo/siGal-9	Decrease gal-9 expression	GBM model in mice	Promotion of macrophage repolarization and restricts the immunosuppression of GBM	Li et al. (2023)
P4D2 mAb	Bind the C-terminal CRD of gal-9	MM model in mice	Tumor growth was inhibited and the infiltration of tumor-associated M2 macrophages was reduced	Bertino et al. (2019)
α-lactose	Blockade of gal-9/Tim-3 signaling	Hsv-infected mice	Effector and memory CD8(+) T cell numbers were increased, resulting in a marked increase in viral control efficiency	Sehrawat et al. (2010)
Anti-gal-9 antibody	Neutralization of gal-9	Tumor model in mice	Synergized with ATM inhibition to induce potent antitumor immunity	Zheng et al. (2023)

## 5 Conclusion

Immune cells play a role in immune surveillance, immune defense and immune homeostasis in the body, and their quantity and quality are closely related to human health. Gal-9 is characterized as a multifunctional protein that is widely distributed in the cells of the innate and adaptive immune system. Gal-9 can directly or indirectly involved in many biological processes during the progression of immune diseases. In different immune cells, gal-9 plays different roles. In the innate immune cells, gal-9 mainly plays an active role in preventing the invasion of pathogenic microorganisms and anti-tumor. However, in adaptive immune cells, gal-9 mainly plays an inhibitory role, which is conducive to the invasion of pathogenic microorganisms and immune escape of tumor cells. Additionally, the regulation of gal-9 on the same immune cell may be beneficial or disadvantageous in different immune diseases. For example, gal-9 can promote immune evasion in HBV-associated HCC cells by inhibiting the immune function of T cells, but improves autoimmune arthritis by regulating Th1/Th2 imbalance.

Therefore, it is of interest to find drugs that can affect the action of gal-9 for the treatment of immune diseases. At present, the inducers or inhibitors of gal-9 inhibitors have been found to regulate the immune function of immune cells by regulating the expression of gal-9, inhibiting the effect of gal-9 by directly binding to CRD, or blocking the binding of gal-9 to Tim-3. These inducers or inhibitors have been shown to be effective in treating some autoimmune diseases *in vivo*.

In conclusion, this review systematically detailed the role of gal-9 in the regulation of different immune cells in innate and adaptive immunity, as well as enumerating several inhibitors and promoters of gal-9. Gal-9 may be a potential target for the treatment of various immune diseases. Even so, the effect of immune cells on the expression of gal-9 and the specific mechanism of gal-9 on autoimmune diseases still need to be further studied. Further study for more effective agents targeting gal-9 may provide new ideas for early diagnosis and precise treatment of autoimmune diseases.
